# Results of the 2018 Japan Society for Blood Purification in Critical Care survey: current status and outcomes

**DOI:** 10.1186/s41100-022-00445-0

**Published:** 2022-11-12

**Authors:** Masanori Abe, Hidetoshi Shiga, Hiroomi Tatsumi, Yoshihiro Endo, Yoshihiko Kikuchi, Yasushi Suzuki, Kent Doi, Taka-Aki Nakada, Hiroyuki Nagafuchi, Noriyuki Hattori, Nobuyuki Hirohashi, Takeshi Moriguchi, Osamu Yamaga, Osamu Nishida

**Affiliations:** 1 The Survey Committee, Japan Society for Blood Purification in Critical Care, Tokyo, Japan; 2 Division of Nephrology, Hypertension and Endocrinology, Department of Medicine, Nihon University School of Medicine, Tokyo, Japan; 3 Emergency and Intensive Care Center, Teikyo University Chiba Medical Center, Ichihara, Chiba Japan; 4 Department of Intensive Care Medicine, Sapporo Medical University School of Medicine, Sapporo, Hokkaido Japan; 5 Shiga University of Medical Science, Otsu, Shiga Japan; 6 Department of Critical Care and Disaster Medicine, Iwate Medical University, Morioka, Iwate Japan; 7 Department of Emergency and Critical Care Medicine, The University of Tokyo, Tokyo, Japan; 8 Department of Emergency and Critical Care Medicine, Chiba University Graduate School of Medicine, Chiba, Japan; 9 Department of Emergency and Critical Care Medicine, Kanagawa Children’s Medical Center, Yokohama, Kanagawa Japan; 10 Department of Radiation Disaster Medicine, Research Institute for Radiation Biology and Medicine, Hiroshima University, Hiroshima, Japan; 11 Department of Emergency and Critical Care Medicine, University of Yamanashi, Graduate School of Medicine, Chuo, Yamanashi Japan; 12 Clinical Engineering Center, Kurume University Hospital, Kurume, Fukuoka Japan; 13 Department of Anesthesiology and Critical Care Medicine, Fujita Health University, Toyoake, Aichi Japan

**Keywords:** Acute kidney injury, Blood purification, Continuous renal replacement therapy, Multiple organ failure, Sepsis

## Abstract

**Background:**

The Japan Society for Blood Purification in Critical Care (JSBPCC) has reported survey results on blood purification therapy (BPT) for critically ill patients in 2005, 2009, and 2013. To clarify the current clinical status, including details of the modes used, treated diseases, and survival rate, we conducted this cohort study using data from the nationwide JSBPCC registry in 2018.

**Methods:**

We analyzed data of 2371 patients who underwent BPT in the intensive care units of 43 facilities to investigate patient characteristics, disease severity, modes of BPTs, including the dose of continuous renal replacement therapy (CRRT) and hemofilters, treated diseases, and the survival rate for each disease. Disease severity was assessed using Acute Physiology and Chronic Health Evaluation (APACHE) II and Sequential Organ Failure Assessment (SOFA) scores.

**Results:**

BPT was performed 2867 times in the 2371 patients. Mean APACHE II and SOFA scores were 23.5 ± 9.4 and 10.0 ± 4.4, respectively. The most frequently used mode of BPT was CRRT (67.4%), followed by intermittent renal replacement therapy (19.1%) and direct hemoperfusion with the polymyxin B-immobilized fiber column (7.3%). The most commonly used anticoagulant was nafamostat mesilate (78.6%). Among all patients, the 28-day survival rate was 61.7%. CRRT was the most commonly used mode for many diseases, including acute kidney injury (AKI), multiple organ failure (MOF), and sepsis. The survival rate decreased according to the severity of AKI (*P* = 0.001). The survival rate was significantly lower in patients with multiple organ failure (MOF) (34.6%) compared with acute lung injury (ALI) (48.0%) and sepsis (58.0%). Multivariate logistic regression analysis revealed that sepsis, ALI, acute liver failure, cardiovascular hypotension, central nervous system disorders, and higher APACHE II scores were significant predictors of higher 28-day mortality.

**Conclusion:**

This large-scale cohort study revealed the current status of BPT in Japan. It was found that CRRT was the most frequently used mode for critically ill patients in Japan and that 28-day survival was lower in those with MOF or sepsis. Further investigations are required to clarify the efficacy of BPT for critically ill patients.

*Trial Registration***:** UMIN000027678.

**Supplementary Information:**

The online version contains supplementary material available at 10.1186/s41100-022-00445-0.

## Introduction

Survival rates for patients admitted to the intensive care unit (ICU) have improved over the past decade [1–3]. Reduced in-hospital mortality has also been reported for patients commonly managed in the ICU, such as those with sepsis, acute lung injury（ALI), or aortic dissection [3–5]. Studies focusing on prognostic systems for ICU patients have also reported decreased in-hospital mortality over the past 20 years [6, 7]. These improvements in the survival of critically ill patients have been attributed to improvements in treatment effectiveness, better care before ICU admission, and more frequent discharge to post-acute care facilities [6, 7]. However, the mortality rate of critically ill patients with severe acute kidney injury (AKI) requiring renal replacement therapy (RRT) remains high, especially when AKI occurs secondary to sepsis [8, 9]. In addition, AKI is often associated with multiple organ failure (MOF), and the mortality rate of AKI is higher when accompanied by sepsis or MOF. RRT is often required for patients with severe AKI, but blood purification therapy (BPT) other than RRT is also an option for some critically ill patients. For patients with sepsis, endotoxin adsorption by direct hemoperfusion with a polymyxin B-immobilized fiber column (PMX-DHP) has been used in Japan since 1994 [10]. Other methods such as apheresis and adsorption are also used in Japan, and new types of hemofilters as that can adsorb cytokines and endotoxins have become available.

The Japan Society for Blood Purification in Critical Care (JSBPCC) has reported survey results for BPT in critical care in Japan in 2005, 2009, and 2013 [11, 12]. In those reports, the mode of BPT, treated diseases, indications for various therapeutic options, and survival rates were investigated**.** JSBPCC conducted this cohort study using a nationwide registry of critically ill patients who were treated with BPT in 2018, with the aim of clarifying its current status, including the number of critically ill patients treated with BPT, the diseases treated, the mode of BPT, and survival rates.

## Methods

### Registry

JSBPCC has created a registry for data from its nationwide surveys of critically ill patients. The details of the methods have been described previously [11, 12]. Briefly, data were collected for 2371 patients treated with BPT in the ICUs of 43 facilities in Japan. The survey period was January 2018 to December 2018.

Data were collected on patients’ demographic and clinical characteristics, including age, sex, medical history, chronic dialysis status, the presence of sepsis, primary and secondary diseases treated with BPT, mode of BPT, dose of blood purification, Sequential Organ Failure Assessment (SOFA) scores [13], Acute Physiology and Chronic Health Evaluation (APACHE) II scores [14], number of organ failures, types of hemofilters, types of anticoagulants at initiation of BPT, and outcome. AKI stage at initiation of BPT was determined according to the KDIGO (Kidney Disease: Improving Global Outcomes) classification [15]. MOF was defined as the failure of ≥ 2 organs. Sepsis was clinically diagnosed based on published consensus criteria [16]. Dates of death during the study period were recorded. The primary outcome was 28-day survival. Exclusion criteria were age < 20 years, chronic dialysis therapy, and missing data on date of birth, mode of BPT, primary disease, or outcome.

This study was conducted in accordance with the Declaration of Helsinki, Japanese privacy protection laws, and the Ethical Guidelines for Medical and Health Research Involving Human Subjects published by the Ministry of Education, Culture, Sports, Science and Technology and the Ministry of Health, Labour and Welfare in 2015. The study was approved by the ethics committee of Chiba University Hospital. No personally identifiable information is stored in the JSBPCC registry. The need for informed consent was waived due to the use of de-identified data. This study is registered with the University Hospital Medical Information Network (UMIN000027678).

### Diseases treated with BPT and modes of BPT in Japan

The following diseases are treated with BPT in Japan: (1) AKI, (2) sepsis, (3) congestive heart failure, (4) MOF, (5) acute liver failure (ALF), (6) acute electrolyte, fluid, and acid–base disorders, (7) acute exacerbation of autoimmune disease, (8) severe acute pancreatitis, (9) ALI, (10) thrombotic microangiopathy, (11) acute drug intoxication, and (12) others, including Guillain–Barré syndrome, toxic epidermal necrolysis, and acute metabolic disorders. The modes of BPT used in Japan are listed in Additional file 1

Primary and secondary diseases requiring BPT and the modes of BPT for these diseases were recorded. For each disease, the number of cases was recorded as the cumulative number because many patients had multiple diseases at initiation of BPT. Similarly, for each mode of BPT, the number of cases was recorded as a cumulative number because some patients were treated with multiple modes.

### Statistical methods

Data are reported as numbers and proportions or as the mean ± standard deviation. Categorical variables were analyzed using the Chi-square test, and continuous variables were compared using the *t*-test. Comparison among three or more groups was performed using repeated-measures analysis of variance with Tukey’s honestly significant difference test or the Kruskal–Wallis test, as appropriate.

For analysis of survival, patients were divided into eight age groups (20–29 years, 30–39 years, …, ≥ 90 years), which were defined a priori. Furthermore, we compared the survival rate according to the primary disease requiring BPT. Among patients with AKI, subgroup analysis was performed according to KDIGO classification (stage 1, 2, or 3) [15] for comparisons of mode of RRT, severity, and survival rate. Furthermore, the survival rate was compared according to hemofilter type between polysulfone (PS), polymethylmethacrylate (PMMA), acrylonitrile-co-methallyl sulfonate surface-treated (AN69ST), and other types of membranes in patients treated with continuous renal replacement therapy (CRRT). Survival analyses using Cox proportional-hazards regression models were performed to examine whether factors at baseline (e.g., age, sex, presence or absence of sepsis, AKI, ALI, ALF, cardiovascular hypotension, coagulation disorders, central nervous system disease, and APACHE II score) predicted 28-day mortality. The PS group was defined as the reference group because the PS hemofilter was the most widely used. To identify independent predictors of 28-day survival, multivariate logistic regression analysis was performed with the following covariates: age, sex, SOFA score, APACHE II score, the presence or absence of AKI, ALI, ALF, sepsis, cardiovascular hypotension, coagulation disorders, and central nervous system disorder. For regression, missing data were imputed using conventional methods, as appropriate. All analyses were performed using JMP® version 13.0 (SAS Institute, Cary, NC), and the level of statistical significance was set as *P* < 0.05.

## Results

### Patient characteristics

**Fig. 1:**
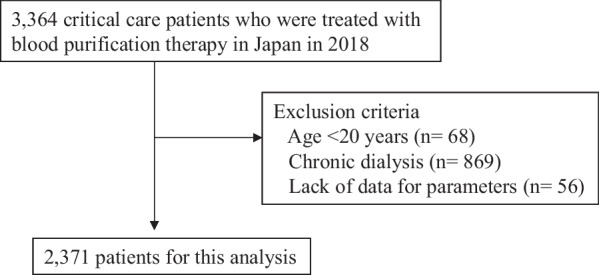
Study flowchart

Overall, 3364 patients were registered and 993 were excluded, leaving 2371 patients for inclusion in the analysis (Fig. 1). Baseline characteristics of the patients are shown in Table 1. Mean age was 68.8 ± 15.2 years, and 33.9% of the patients were female. In terms of disease severity, mean SOFA score was 10.0 ± 4.4, and mean APACHE II score was 23.5 ± 9.4. In total, 2867 BPT procedures were performed. The most frequently used mode was CRRT (67.4%), especially continuous hemodiafiltration (CHDF) (48.0%), followed by intermittent renal replacement therapy (IRRT) (19.1%) and continuous hemodialysis (CHD) (13.3%). Per patient, the mean number of modes of BPT was 1.2 ± 0.5, and most patients (86.1%) were treated with a single mode of BPT. The most commonly used anticoagulant at initiation of BPT was nafamostat mesilate (78.6%), followed by heparin (15.6%). During the 1-year study period, 909 patients died (38.3%), and the 28-day survival rate was 61.7% (1462 patients alive).

**Table 1:** Demographic and clinical characteristics of the patients

Variable	
Patients treated with BPT (*n*)	2371
Sex (% female)	33.9
Age (years)	68.8 ± 15.2
Sepsis (%)	40.8
APACHE II score	23.5 ± 9.4
SOFA score	10.0 ± 4.4
Mortality	38.3%
Total number of BPT procedures performed (*n*)	2867
Details of BPT [*n* (%)]	
Continuous renal replacement therapy	1932 (67.4)
Continuous hemodiafiltration	1376 (48.0)
Continuous hemodialysis	382 (13.3)
Continuous hemofiltration	174 (6.1)
Sustained low-efficiency hemodialysis	28 (1.0)
Intermittent renal replacement therapy	547 (19.1)
Direct hemoperfusion with PMX	209 (7.3)
Direct hemoperfusion with activated carbon	4 (0.1)
Simple plasma exchange	126 (4.4)
Double filtration plasmapheresis	6 (0.2)
Plasma adsorption	12 (0.4)
Others	3 (0.1)
Number of modes of BPT per patient [*n* (%)]	
1	2041 (86.1)
2	263 (11.1)
3	54 (2.3)
≥ 4	13 (0.5)
Anticoagulants [*n* (%)]	
Nafamostat mesilate	1864 (78.6)
Heparin	370 (15.6)
Low molecular weight heparin	14 (0.6)
None	116 (4.9)
Others	7 (0.3)

*APACHE* Acute Physiology and Chronic Health Evaluation, *BPT* blood purification therapy, *PMX* polymyxin B-immobilized fiber column, *SOFA* Sequential Organ Failure Assessment

### Mode of BPT for each disease

The modes of BPT used for each disease are listed in Table 2. For AKI, the two most frequently used modes were CHDF (50.0%) and IRRT (23.8%). For sepsis, CHDF (48.2%) and PMX-DHP (27.5%) were the most frequently used. For congestive heart failure, MOF, acute electrolyte disorders, and severe acute pancreatitis, CHDF was most frequently used (66.7%, 40.0%, 43.1%, and 79.5%, respectively). For ALF, the most frequently used modes were simple plasma exchange (SPE; 40.0%) and CHDF (31.1%). For ALI, CHDF was the most common (34.0%), followed by IRRT (20.8%) and PMX-DHP (12.3%). SPE was the most frequently used mode for both autoimmune diseases (48.1%) and thrombotic microangiopathy (59.4%).

**Table 2:** Mode of blood purification therapy according to disease

	Acute kidney injury	Sepsis	Congestive heart failure	Multiple organ failure	Acute liver failure	Acute electrolyte disorders	Autoimmune diseases	Severe acute pancreatitis	Acute lung injury	Thrombotic microangiopathy	Acute drug intoxication
Cumulative number of patients	1182	680	336	190	90	137	52	44	106	32	18
CRRT	880 (74.4)	418 (61.5)	276 (82.1)	113 (59.5)	36 (40.0)	86 (62.8)	8 (15.4)	40 (90.9)	58 (54.7)	6 (18.8)	11 (61.1)
CHDF	579 (50.0)	328 (48.2)	224 (66.7)	76 (40.0)	28 (31.1)	59 (43.1)	3 (5.8)	35 (79.5)	36 (34.0)	4 (12.5)	4 (22.2)
CHD	209 (17.7)	53 (7.8)	41 (12.2)	27 (14.2)	7 (7.8)	18 (13.1)	1 (1.9)	1 (2.3)	16 (15.1)	2 (6.3)	7 (38.9)
CHF	92 (7.8)	37 (5.4)	11 (3.3)	10 (5.3)	1 (1.1)	9 (6.6)	4 (7.7)	4 (9.1)	6 (5.7)	0 (0)	0 (0)
SLED	21 (1.8)	1 (0.1)	2 (0.6)	1 (0.5)	1 (1.1)	0 (0)	0 (0)	0 (0)	2 (1.9)	0 (0)	0 (0)
IRRT	281 (23.8)	73	58 (17.2)	32 (16.8)	11 (12.2)	51 (37.2)	5 (9.6)	4 (9.1)	22 (20.8)	7 (21.9)	3 (16.7)
Direct hemoperfusion with PMX	0 (0)	188 (27.5)	0 (0)	8 (4.2)	0 (0)	0 (0)	0 (0)	0 (0)	13 (12.3)	0 (0)	0 (0)
Direct hemoperfusion with activated carbon	0 (0)	0 (0)	0 (0)	0 (0)	0 (0)	0 (0)	0 (0)	0 (0)	0 (0)	0 (0)	4 (22.2)
Simple plasma exchange	0 (0)	0 (0)	0 (0)	35 (18.4)	36 (40.0)	0 (0)	25 (48.1)	0 (0)	11 (10.4)	19 (59.4)	0 (0)
Double filtration plasmapheresis	0 (0)	0 (0)	0 (0)	0 (0)	0 (0)	0 (0)	6 (11.5)	0 (0)	0 (0)	0 (0)	0 (0)
Plasma adsorption	0 (0)	0 (0)	0 (0)	1 (0.5)	3 (3.3)	0 (0)	8 (15.4)	0 (0)	0 (0)	0 (0)	0 (0)
Others	0 (0)	0 (0)	0 (0)	0 (0)	3 (3.3)	0 (0)	0 (0)	0 (0)	0 (0)	0 (0)	0 (0)

Data are expressed as n or n (%). CHF, continuous hemofiltration; CHD, continuous hemodialysis, CHDF, continuous hemodiafiltration; CRRT, continuous renal replacement therapy; PMX, polymyxin B-immobilized fiber column; IRRT, intermittent renal replacement therapy; SLED, sustained low-efficiency hemodialysis. Continuous renal replacement therapy, typically performed for 24 h, is divided into continuous hemodiafiltration, continuous hemodialysis, and continuous hemofiltration. Intermittent renal replacement therapy, typically performed in 4-h session 3 times weekly, includes intermittent hemodialysis or intermittent hemodiafiltration. Direct hemoperfusion with PMX is indicated for endotoxemia or sepsis due to gram-negative bacillus as well as severe sepsis. Direct hemoperfusion with activated carbon is mainly indicated for acute drug intoxication. Double filtration plasmapheresis and plasma adsorption are indicated in autoimmune diseases for removal or adsorption of autoantibodies. Simple plasma exchange is indicated in liver failure, autoimmune diseases, HUS, TTP, Guillain–Barré syndrome, and toxic epidermal necrolysis, among other conditions

### Survival rate

**Fig. 2:**
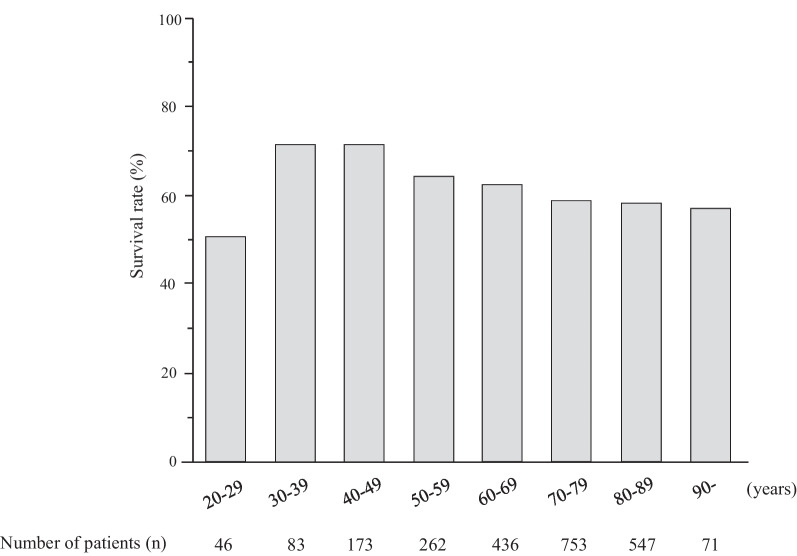
Survival rate according to age group. There was no significant difference in the survival rate among the age groups (*P* = 0.273)

**Fig. 3:**
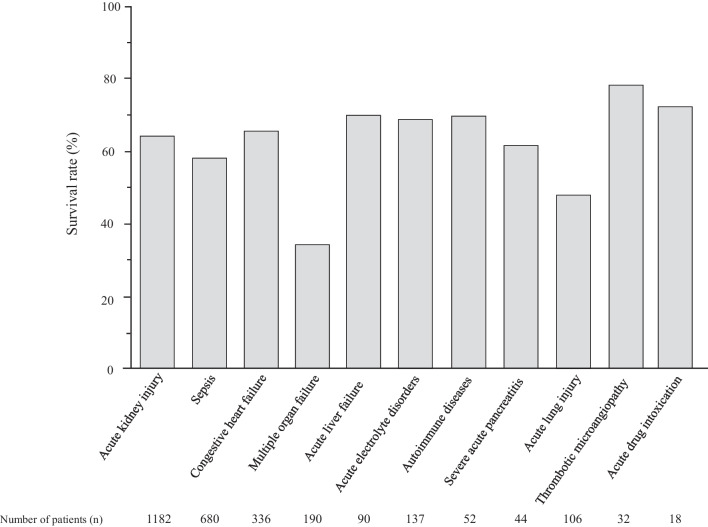
Survival rate in patients with different diseases. There was a significant difference in 28-day survival rate among the diseases (P for trend < 0.0001)

Figure 2 shows the 28-day survival rate according to 10-year age group. No significant differences was noted among the groups (*P* = 0.273). By disease, 28-day survival was significantly lower in patients with MOF (34.6%), ALI (48.0%), and sepsis (58.0%) (*P* < 0.0001) (Fig. 3).

### Efficacy and survival rate in AKI

Of all 2371 patients, 2140 (90.3%) were diagnosed as having AKI based on the KDIGO criteria. In a subgroup analysis according to AKI stage (stage 1, 2, or 3), 70.7% of these 2140 patients had stage 3 AKI (Table 3). Age and sex did not significantly differ according to AKI stage, though APACHE II and SOFA scores were significantly lower in stage 1 AKI compared with stages 2 and 3. The comorbidity rate for sepsis and the frequency of PMX-DHP use were significantly different among these three subgroups. There was significantly lower 28-day survival according to the severity of the AKI stage.

**Table 3:** Comparison of characteristics and 28-day survival rate according to AKI stage

	AKI stage 1	AKI stage 2	AKI stage 3	*P* value
Number of patients [*n* (%)]	212	416	1512	
Sex (% female)	39.2	31.3	33.1	0.129
Age (years)	70.0 ± 14.3	69.4 ± 15.3	69.1 ± 14.7	0.632
APACHE II score	22.2 ± 9.2	23.0 ± 8.8	24.4 ± 9.2	0.0001
SOFA score	8.7 ± 3.9	9.9 ± 4.2	10.7 ± 4.3	< 0.0001
Comorbid sepsis [*n* (%)]	51.4	43.0	40.0	0.005
Renal replacement therapy [*n* (%)]	192 (90.5)	388 (93.2)	1459 (96.6)	< 0.0001
CRRT [*n* (%)]	136 (70.8)	318 (82.0)	1134 (77.7)	
IRRT [*n* (%)]	49 (25.5)	42 (10.8)	226 (15.5)	
Both CRRT and IRRT [*n* (%)]	7 (3.7)	28 (7.2)	99 (6.8)	
PMX-DHP [*n* (%)]	28 (13.2)	41 (9.9)	99 (6.5)	0.001
Survival rate (%)	68.9	63.5	58.8	0.001

*AKI* acute kidney injury, *APACHE* Acute Physiology and Chronic Health Evaluation, *CRRT* continuous renal replacement therapy, *IRRT* intermittent renal replacement therapy, *PMX-DHP* direct hemoperfusion with polymyxin B-immobilized fiber column

### Details of CRRT

The dose of blood purification for CRRT was 17.9 ± 8.6 mL/kg/h. In patients treated with CRRT, the membranes used were PS, PMMA, AN69ST, and other types in 36.2%, 23.6%, 22.1%, and 18.1%, respectively (Table 4). The 28-day survival was significantly higher for the other types of membranes compared with PS, PMMA, and AN69ST (*P* < 0.0001). The hazard ratio (HR) of the PMMA group was significantly higher and that of other types of membrane group was significantly lower compared with the PS (reference) group. However, the significance was lost after adjustment for characteristics at baseline (Table 5).

**Table 4:** Types of hemofilters and survival rate

Hemofilters	PS	PMMA	AN69ST	Others	*P* value
*n* (%)	675 (36.2)	440 (23.6)	412 (22.1)	337 (18.1)	–
28-day survival rate (%)	58.5	52.7	54.1	70.8	< 0.0001

*AN69ST* acrylonitrile-co-methallyl sulfonate surface-treated, *PMMA* polymethylmethacrylate, *PS* polysulfone

**Table 5:** Hazard ratios (with 95% confidence intervals) for mortality according to hemofilter

Hemofilters	*n*	Unadjusted			Adjusted for baseline characteristics		
		HR	95%CI	*P* value	HR	95%CI	*P* value
PS	675	1.00	Reference	–	1.00	Reference	–
PMMA	440	1.29	1.01–1.64	0.039	1.14	0.91–1.43	0.225
AN69ST	412	1.19	0.93–1.53	0.156	0.95	0.77–1.17	0.638
Others	337	0.58	0.43–0.77	0.001	0.85	0.63–1.12	0.265

HRs for 28-day mortality in patients who were treated with continuous renal replacement therapy according to hemofilters, determined using standard Cox proportional-hazards regression analysis and adjusted for characteristics at baseline (age, sex, presence or absence of sepsis, acute kidney injury, acute lung injury, acute liver failure, cardiovascular hypotension, coagulation disorders, central nervous system disease, and APACHE II score). *AN69ST* acrylonitrile-co-methallyl sulfonate surface-treated, *APACHE* Acute Physiology and Chronic Health Evaluation, *CI* confidence interval, *HR* hazard ratio, *PMMA* polymethylmethacrylate, *PS* polysulfone

### Multivariate logistic regression analysis

Multivariate logistic regression analysis with stepwise entry was performed to identify independent predictors of 28-day survival in critically ill patients (Table 6). The response variable was 28-day survival and the covariates were age, sex, APACHE II score, and the presence of AKI, ALI, ALF, sepsis, cardiovascular hypotension, coagulation disorders, and central nervous system disorders. APACHE II score and the presence of sepsis, ALI, ALF, cardiovascular hypotension, and central nervous system disorder were identified as being significantly associated with lower 28-day survival rate.

**Table 6:** Multivariate logistic regression analysis of determinants of 28-day survival

Variables	Estimate	SE	95%CI		*P* value
			Lower	Upper	
Age (years)	0.007	0.004	− 0.0002	0.014	0.057
Sex (male)	0.017	0.054	− 0.089	0.123	0.751
Sepsis (yes)	0.128	0.052	0.025	0.123	0.014
APACHE II score	0.055	0.006	0.042	0.067	< 0.0001
Acute kidney injury (yes)	0.104	0.058	− 0.008	0.219	0.072
Acute lung injury (yes)	0.145	0.069	0.011	0.281	0.035
Acute liver failure (yes)	0.258	0.054	0.152	0.364	< 0.0001
Coagulation disorders (yes)	0.089	0.056	− 0.021	0.2	0.113
Cardiovascular hypotension (yes)	0.235	0.061	0.117	0.354	0.0001
Central nervous system disorders (yes)	0.243	0.061	0.131	0.354	< 0.0001

*APACHE* Acute physiology and chronic health evaluation, *CI* confidential interval, *SE* standard error

## Discussion

In this 2018 survey, the total number of critically ill patients who were treated with any kind of BPT was 2371. Total number of BPT procedures was 2867 because some patients received more than two modes of therapies. Given that this survey covered only 43 hospitals, the actual number of patients nationwide in Japan who were treated with BPT would be much larger than the size of this cohort. The most frequently used mode was CRRT (67.4%), and CHDF in particularly accounted for 48.0% of all BPT procedures. The next most common was intermittent renal replacement therapy (19.1%). SOFA score, which could not be investigated in the 2013 survey, was used to examine the effect of organ failure on 28-day survival in this cohort. Multivariate logistic regression analysis revealed that the presence of sepsis, ALI, ALF, cardiovascular hypotension, and central nervous system disorders in addition to higher APACHE II scores were significant predictors of lower 28-day survival in the 2018 survey.

The JAKID study investigated the diagnosis, treatment, and prognosis of 2292 patients who were admitted to the ICU from June to December 2016 at 13 hospitals in Japan [17]. Among those 2292 patients, AKI was diagnosed in 1024 patients (44.7%), and RRT was used to treat 171 patients (16.7% of patients with AKI) during the ICU stay. Furthermore, RRT was performed for 30 patients with non-AKI. CHDF (45%) was the most common mode of RRT, followed by CHD (25.6%), and IRRT (11.6%). The in-hospital mortality rate among patients who received RRT for AKI in Japan during the period 2007–2016 has been reported based on data from the Diagnosis Procedure Combination database [18]. In total, 39,471 patients (76.3%) were treated with CRRT and 12,287 patients (23.7%) were treated with IRRT. The adjusted odds ratio (OR) for in-hospital mortality was 0.66 (95% confidence interval [CI] 0.60–0.72) in 2016 compared to 2017, with a downward trend observed for both patients starting CRRT (adjusted OR 0.67, 95% CI 0.61–0.75) and those starting IRRT (0.58, 0.45–0.74). Furthermore, mortality decreased in all age groups. In recent years, many reports have shown that the mortality rate of critically ill patients has decreased [1–3, 19, 20]. Despite increases in patient age and disease severity, a relative 35% reduction in mortality among patients admitted to the ICU from 1988 to 2012 was found in the United States [3]. Also, age was not associated with mortality in the present cohort, the same as in the 2013 survey. The reason for the decrease in mortality may be that RRT has been used for less severe AKI. It has been reported that RRT tends to be used for non-renal indications in Japan [18]. Therefore, AKI might not be associated with 28-day survival rate in this cohort. In addition, the present cohort was characterized by a higher prevalence of patients who required RRT even though they had AKI stage 1 and by a higher prevalence of sepsis in patients with stage 1 AKI. Accordingly, CRRT using hemofilters with cytokine-adsorbing ability, including PMMA and AN69ST, has been used as one of the therapeutic options for sepsis in Japan. Furthermore, RRT is also used to treat other diseases, such as congestive heart failure, where AKI is not the primary therapeutic target. Thus, patients with early stages of AKI might have been included in this cohort.

RRT is the mainstay of treatment for severe AKI. For dialysis in the ICU, CRRT has primarily been used because of its accurate volume control, acid–base stability, and electrolyte correction, as well as its ability to achieve hemodynamic stability. CRRT can improve the clearance rate of small, medium, and large molecules in blood by removing water via ultrafiltration and making hemodynamics more stable. Small molecule clearance rates are low, and hemofiltration requires large volumes of fluid replacement. In the previous JSBPCC surveys conducted in 2005, 2009, and 2013, the frequency of CHDF was 50.3%, 53.0%, and 50.6%, respectively [11, 12]. In the present study, CHDF was provided to 48.0% of the patients, showing a decreasing trend compared with 2009 and 2013. On the other hand, the frequency of CHD showed a marked increase, reaching 13.5% in 2018 compared with 5.5% in 2013. The CHD mode contributes to prolonging the membrane lifetime of the hemofilter without increasing the transmembrane pressure compared with filtration mode. Although inflammatory cytokines and mediators are conventionally removed by convection, cytokine-adsorbing hemofilter can remove them without convection and are widely used in Japan. As a result, the frequency of CRRT in 2018 was 67.4% overall, demonstrating an upward trend for continuous therapy.

The frequency of using PMX-DHP is declining. It was performed in 15.0% of patients in the 2005 and 2009 surveys and 11.5% in the 2013 survey [11, 12]. In the present survey, a further decrease to 7.5% was observed. In the EUPHRATES randomized clinical trial reported in 2018, 450 patients who had septic shock with an endotoxin activity assay (EAA) value ≥ 0.60 were assigned to a PMX-DHP group (*n* = 224) and a sham hemoperfusion group (*n* = 226) [21]. No significant difference in survival was found between the two groups. However, a sub-analysis of this trial showed that the PMX-DHP group had significantly increased mean arterial blood pressure at 72 h, reduced days on mechanical ventilation, and reduced days on RRT in patients with moderate disease severity who had a high Multiple Organ Dysfunction Score (> 9) and high EAA (0.6 ≤ EAA < 0.9) [22]. Furthermore, in a propensity score-matched study comparing a PMX group and non-treated group in patients with septic shock who were treated with noradrenaline [23], 28-day survival was significantly better in the PMX group compared with the non-treated group (*P* < 0.0001), and median days of noradrenalin treatment, days of CHDF treatment, and ventilator-free days were significantly higher in the PMX group. However, considering the conflicting results and the low quality of evidence, Surviving Sepsis Campaign 2021 and The Japanese Clinical Practice Guidelines for Management of Sepsis and Septic Shock 2020 recommend against using PMX-DHP for patients with septic shock [24, 25].

PS membranes and AN69ST membranes are now frequently used as hemofilters for CRRT outside of Japan, PMMA was frequently used in the 2013 JSBPCC survey in Japan [12]. However, the AN69ST hemofilter was not introduced in Japan until 2014, so the frequency of using AN69ST membranes has naturally increased since the previous survey. In Japan, focus has been placed on cytokine removal by adsorption for patients with hypercytokinemia due to sepsis or other conditions. CHDF using a cytokine-adsorbing hemofilter made from a PMMA or AN69ST membrane can continuously and effectively remove many kinds of inflammatory cytokines and decrease their blood levels [26–29]. On the other hand, in other countries focus has been placed on cytokine removal by filtration using high cut-off membranes and medium cut-off membranes [30, 31]. However, these membranes are not marketed in Japan. Despite reports that PMX-DHP was effective in patients with sepsis who were treated with CRRT [32], AN69ST membranes can reduce both cytokine and endotoxin levels in patients with septic shock [33, 34]. Furthermore, AN69ST membranes are used in patients with sepsis with or without AKI in Japan. Therefore, the number of patients treated with PMX-DHP might have decreased due to the increased use of AN69ST membranes in this cohort. However, we did not find any difference in survival between AN69ST membranes and other types of hemofilter in the present cohort. Further research is needed to clarify what type of hemofilter can best improve prognosis.

The optimal dose of CRRT is not clear. For patients with AKI, the KDIGO recommendations are weekly Kt/V of 3.9 when using intermittent or extended RRT and an effluent volume of 20–25 ml/kg/h for CRRT [15]. This will usually require a prescription of a higher effluent volume. In clinical practice in Japan, however, the dose of CRRT is lower from that used in the United States and other countries. The approved dose of sterile dialysis fluid or substitution fluid is up to 14–15 L daily, and correspondingly the mean dose of CRRT in the present cohort was 18.7 ± 12.5 mL/kg/h, which was nearly equivalent to the dose in the 2013 cohort. Furthermore, CRRT at a mean intensity of 14.3 mL/kg/h, the standard dose in Japan, was found to be non-inferior to 20–25 mL/kg/h of CRRT, which is currently considered the standard intensity outside of Japan [35]. Our results showing favorable survival despite the lower dose of CRRT in the present cohort are consistent with that report. The number of HDF patients in Japan has rapidly increased since 2012. Facility survey data at the end of 2018 has shown that 38.3% of all patients on chronic hemodialysis therapy received on-line HDF therapy [36]. Accordingly, some facilities offer on-line HDF treatment for critically ill patients in Japan.

This study has some limitations that should be kept in mind. First, observational cohort studies and repeated surveys conducted every few years may have variations in mortality between centers due to changes in the practices and patient populations of each center. In addition, BPT for critically ill patients was not performed according to the same protocol. Variations in the therapeutic regimen may have affected the responses to therapy and outcomes. However, we consider the present results to be representative of the actual clinical setting for BPT in Japan because our survey was conducted nationwide at 43 centers in various urban and rural locations. Second, data were not available for some possible confounders, such as residual kidney function, diabetes status, and serum albumin levels, but it is known that diabetes and hypoalbuminemia are associated with mortality in patients with AKI [37, 38]. Third, the study period was before the coronavirus disease 2019 (COVID-19) pandemic, so COVID-19 cases were not included. COVID-19 causes not only pneumonia but also MOF including various renal complications, and it has been reported that prognosis is poorer in AKI patients with COVID-19 than in non-AKI patients with COVID-19 [39]. Furthermore, filter life tends to be shortened due to thrombophilia in severe COVID-19 [40, 41]. Further studies are needed to determine whether cytokine removal by adsorption or filtration in CRRT is effective in patients with COVID-19.

In conclusion, this study has revealed that CRRT, especially the CHDF mode, was most frequently used among critically ill patients in Japan. The 28-day survival rate of patients with AKI was relatively high, but lower in cases with MOF such as ALI or ALF or cases with sepsis.

## Supplementary Information

**Additional file 1. Supplementary Table 1.** Modalities of blood purification therapy currently used in Japan and their common abbreviations

## Data Availability

The data used in this article are available from the corresponding author.

## Abbreviations

ALF: Acute liver failure
AKI: Acute kidney injury
ALI: Acute lung injury
AN69ST: Acrylonitrile-co-methallyl sulfonate surface-treated
APACHE: Acute Physiology and Chronic Health Evaluation
BPT: Blood purification therapy
CHDF: Continuous hemodiafiltration
CHD: Continuous hemodialysis
CHF: Continuous hemofiltration
CI: Confidence interval
COVID-19: Coronavirus disease 2019
CRRT: Continuous renal replacement therapy
EAA: Endotoxin activity assay
HDF: Hemodiafiltration
ICU: Intensive care unit
IRRT: Intermittent renal replacement therapy
JSBPCC: Japan Society for Blood Purification in Critical Care
KDIGO: Kidney Disease: Improving Global Outcomes
MOF: Multiple organ failure
PMX-DHP: Direct hemoperfusion with polymyxin B-immobilized fiber column
PMMA: Polymethylmethacrylate
PS: Polysulfone
RRT: Renal replacement therapy
SOFA: Sequential Organ Failure Assessment
