# Supplementary material for: Results of the 2018 Japan Society for Blood Purification in Critical Care survey: current status and outcomes
Source: Ren Replace Ther. 2022 Nov 12;8(1):58. doi: 10.1186/s41100-022-00445-0 (PMC9660154; doi:10.1186/s41100-022-00445-0)
Supplement: Supplementary file 1 — Additional file 1. Supplementary Table 1. Modalities of blood purification therapy currently used in Japan and their common abbreviations [file 41100_2022_445_MOESM1_ESM.docx]

Supplementary Table 1. Modalities of blood purification therapy currently used in Japan and their common abbreviations

| 1. Continuous renal replacement therapy | CRRT |
| --- | --- |
| Continuous hemodiafiltration | CHDF |
| Continuous hemodialysis | CHD |
| Continuous hemofiltration | CHF |
| Extracorporeal ultrafiltraion | ECUM |
| 2. Intermittent renal replacement therapy | IRRT |
| Henodiafiltration | HDF |
| Hemofiltration | HF |
| Hemodialysis | HD |
| Extracorporeal ultrafiltraion | ECUM |
| 3. Sustained low-efficiency hemodialysis | SLED |
| 4. Plasma exchange | PE |
| Simple plasma exchange | SPE |
| Double filtration plasmapheresis | DFPP |
| Plasma filtration with dialysis | PDF |
| 5. Direct hemoperfusion | DHP |
| Direct hemoperfusion with polymyxin B-immobilized fiber column | PMX-DHP |
| Direct hemoperfusion with activated carbon | AC-DHP |
| Plasma adsorption | PA |
